# Clinical Experience with Formaldehyde

**Published:** 1897-09

**Authors:** 


### Abstracts and Selections.

### Clinical Experience with Formaldehyde.

In the *New York Medical Journal*, January, Dr. M. S. Alexander states that he has used this drug in whooping cough and numerous other conditions with gratifying results. It should be remembered that formalin solution has no caustic properties. It does not hurt the instruments not even as much as blunting the edge of the cutting instruments. The same solution was always used for irrigation when the dressing of wounds was changed; this too gave such satisfaction, that it has been altogether used in a great many operations. "I have used it altogether in my practice for almost a year. I have no more need for corrosive sublimate or carbolic acid. I wish to emphasize the fact that formalin does not attack metals at all. It is best adapted for antiseptic solutions (1 to 500) in which to keep instruments. I have had no alarming results with formalin locally; indeed, in cases of chancroid and chancre, the pure (40 per cent.) was applied without any bad results other than pain, with the benefit of rapidly healing the ulcers with a single application. I have never failed to get better results from it in skin troubles than from any other remedy. I used it by blowing it into the skin by a compressed air sprayer.

"I have cured an old case of pruritus vulvæ that had been treated by many different specialists for three years. In all affections when any of the old disinfectants, such as carbolic acid, corrosive sublimate and many others cause trouble by irritation and poison if not applied very carefully, this is all lost sight of and overcome by the happy and safe use of formalin.

"Cases of whooping cough are treated successfully by spraying with an atomizer three times daily, using a 1 per cent. solution for fifteen minutes. I have never had an opportunity to use it in scarlet fever, but no doubt it would prove of great utility in all infectious and contagious diseases for the following reasons: 1. It has extraordinarily active antiseptic power, similar to that of corrosive sublimate; 2. It attacks only the substances of the contagious material, leaving unattacked the articles treated, whether organic or inorganic; 3. It is very readily employed under all circumstances, either as a liquid or as a gas. Another marked advantage of the vapor of formalin is that its specific gravity closely approximates that of air, so that there is no difficulty in keeping the atmosphere of an inclosed space uniformly impregnated. For this reason it suggested itself to me as a happy remedy in catarrhal troubles. In hay fever it surpasses all other agents known. I use a 0.5 per cent. solution, and allowing the patient to inhale the fumes, I can come as near curing the most obstinate forms of catarrhal troubles as by all other means known. The fumes seem to reach portions that cannot be penetrated by the spray or douche. I treated fifteen cases of infantile diarrhea with only one death. Half a grain and a quarter at a dose every two hours. In conclusion I will say that I can clinically recommend formalin as being in the lead among intestinal antiseptics, and far superior to all other agents known in its sphere."

